# Protocol for sleep analysis in the brain of genetically modified adult mice

**DOI:** 10.1016/j.xpro.2021.100982

**Published:** 2021-12-08

**Authors:** Kanako Iwasaki, Noriko Hotta-Hirashima, Hiromasa Funato, Masashi Yanagisawa

**Affiliations:** 1 International Institute for Integrative Sleep Medicine, University of Tsukuba, Tsukuba, Ibaraki 305-8575, Japan; 2 Department of Anatomy, Graduate School of Medicine, Toho University, Ota-ku, Tokyo 951-8585, Japan; 3 Department of Molecular Genetics, University of Texas Southwestern Medical Center, Dallas, TX 75390, USA; 4 Life Science Center, Tsukuba Advanced Research Alliance, University of Tsukuba, Tsukuba, Ibaraki 305-8577, Japan

**Keywords:** Model Organisms, Molecular Biology, Neuroscience, Behavior

## Abstract

Elucidating the molecular pathways that regulate animal behavior such as sleep is essential for understanding how the brain works. However, to examine how a certain functional domain of protein is involved in animal behavior is challenging. Here, we present a protocol for inducing endogenous protein that lacks a specific functional domain using Cre-mediated allele modification in neurons followed by electroencephalogram/electromyogram (EEG/EMG) recording to study the role of kinases in sleep. This strategy is applicable to other gene targets or behaviors.

For complete details on the use and execution of this protocol, please refer to Iwasaki et al. (2021).

## Highlights

• Temporal- and cell type-specific allele induction to regulate protein function
• Intraperitoneal administration of tamoxifen induces Cre-dependent allele induction
• An electrode implant and tether cable acquire EEG/EMG signals of a freely moving mouse
• Allele-specific antibodies show tamoxifen-dependent recombination

## Graphical abstract

**Figure:**
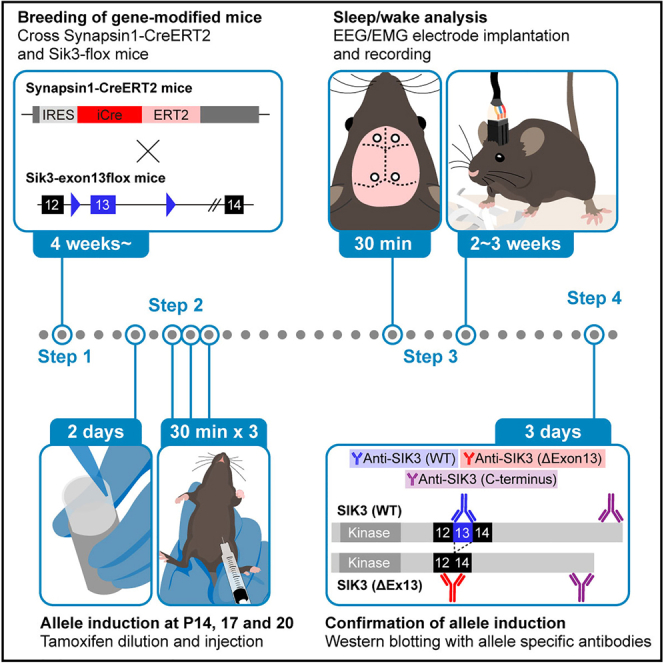

## Before you begin

***Note:*** All animal work presented here was approved (Protocol#180094) and conducted in accordance with the guidelines of the Institutional Animal Care and Use Committee of the University of Tsukuba.

Here, we describe materials and methods for the induction of SIK3 lacking a protein kinase A (PKA)-phosphorylation site in neurons using *Sik3*^*ex13flox*^ and *synapsin1*^*CreERT2*^ mice (Funato et al., 2016; Honda et al., 2018; Iwasaki et al., 2021). Intraperitoneal injection of tamoxifen in late infancy leads to the expected recombination and increases non-rapid eye movement sleep (NREM sleep) in *synapsin1*^*CreERT2*^*; Sik3*^*ex13flox*^ mice. Using other flox mice, the researchers are able to induce or delete the target domain or the entire protein in adult brains. To confirm the successful allele induction, it is desirable to obtain or create a variety of antibodies that detect allele-specific and/or general form of the target protein. When there are no available antibodies, RT-PCR can be used to confirm allele induction. To visualize Cre-mediated recombination induced by tamoxifen, crosses with reporter mouse lines such as *ROSA26*^*LacZ*^ mouse are useful to observe detailed spatial recombination.

### Tamoxifen dosage

In general, higher doses of tamoxifen administration guarantee higher recombination rates, but at the same time lower survival rates (Table 1). Donocoff and colleagues showed that almost all C57BL/6 mouse given five daily 6 mg tamoxifen (equivalent to 200 mg/kg body weight (BW) in a 30 g mouse) were dead in 2 weeks whereas 70% of those given 3 mg tamoxifen survived (Donocoff et al., 2020). According to Valny and colleagues, peak concentrations of tamoxifen metabolite 4-hydroxytamoxifen in adult brains, which presumably be a major CreERT2 inducer, were unchanged between 2 daily 200 mg/kg BW and 100 mg/kg BW administration, but faster degradation was seen at the lower dose (Valny et al., 2016). After five daily intraperitoneal injection of 100 mg/kg BW tamoxifen from P28 to sodium-dependent L-glutamate/L-aspartate transporter (GLAST)-CreERT2 mice results in nearly 100% recombination in the cerebral cortex (Jahn et al., 2018). Five times 100 mg/kg BW tamoxifen injection were performed every alternative day for 10 days from 3 weeks old, which provided high efficient recombination (Huang et al., 2018). These reports suggest that repeated injections of 100 mg/kg BW tamoxifen are reasonable to achieve high recombination efficiency while maintaining low lethality.

**Table 1:** Literature using tamoxifen administration targeting the central nervous system

Tamoxifen administration to neonatal-adolescent mice						
References	PMID	Route	Dose	Timing	Mouse line	Efficiency
(Chen et al., 2009)	19117051	IP to lactating mother	83.5 mg/kg body weight (BW)	Once a day for 5 consecutive days, starting from the day the pups are P0 or P7	Nestin73-CreERT2; Rosa26-LSL(loxP-Stop-loxP)-LacZ	X-gal labeling was observed in the whole cerebellum
(Chen et al., 2009)	19117051	IP	83.5 mg/kg BW	Twice a day for 5 consecutive days in 4- or 8-week old mice	Nestin73-CreERT2; Rosa26-LSL-EYFP, Nestin73-CreERT2; Rosa26-LSL-LacZ	75 ± 4% of Sox2-positive cells in the subventricular zone have been targeted in mice injected tamoxifen from 4-weeks old.
Pitulescu et al. (2010)	20725067	IP	50 μg (25 mg/kg BW in 2 g mouse)	Once a day for 3 consecutive days from P1	Cdh5-CreERT2; Efnb-flox; ROSA26-LSL-EYFP	Robust Cre-mediated recombination was observed in IB4-positive retinal vasculature
Pitulescu et al. (2010)	20725067	IP	100 μg (33 mg/kg BW in 3 g mouse)	Once a day for 4 consecutive days from P5	Cdh5-CreERT2; Efnb-flox; ROSA26-LSL-EYFP	N/A
Pitulescu et al. (2010)	20725067	IP	500 μg (16 mg/kg in 30 g mouse)	Once a day for 5 consecutive days in adult (>8 weeks)	Cdh5-CreERT2; Efnb-flox; ROSA26-LSL-EYFP	N/A
(Petrik et al., 2013)	23991155	IP to lactating mother	150 mg/kg BW	Once a day for 3 consecutive days from P2	Nestin-CreERT2; Rosa26-LSL-EYFP	60% of pups showed YFP-positive cells in the brain
(Petrik et al., 2013)	23991155	IP to lactating mother and PO to pups	150 mg/kg BW	Once a day for 3 consecutive days from P2	Nestin-CreERT2; Rosa26-LSL-EYFP	100% of pups showed YFP-positive cells in the brain
(Huang et al., 2014)	24578301	IP to lactating mother	100 mg/kg BW	Once a day for 2 consecutive days from P3 or P8	NG2-CreERT2; Rosa26-LSL-tdTomato	A large number of tdTomato-positive glia appeared in all brain regions
(Huang et al., 2014)	24578301	IP	1.2 mg in the morning and 1.5 mg 8 h later (40 mg/kg BW and 50 mg/kg BW in 30 g mouse each)	Twice a day for 5 consecutive days in young adult	NG2-CreERT2; Rosa26-LSL-EYFP	∼75% of NG2-positive glia were labeled by EYFP in dorsal cortex or corpus callosum in Rosa26-EYFP mice
(Huang et al., 2014)	24578301	IP	100 mg/kg BW	Once a day for 5 consecutive days in young adult and aged	NG2-CreERT2; Rosa26-LSL-tdTomato	∼95% of NG2-positive glia were labeled by tdTomato in dorsal cortex or corpus callosum in Rosa26-tdTomato mice
(Pohlkamp et al., 2014)	24950299	IP	300 μg (150 mg/kg BW in 2 g mouse)	Once at P3	Nse-CreERT2; ROSA26-LSL-LacZ, Nse-CreERT2; ROSA26-tdTomato-flox-EGFP	Robust Cre-mediated recombination was observed in cerebellar granule cells. Efficiency was not quantified since granule cells are very tiny and densely packed.
(Pohlkamp et al., 2014)	24950299	IP	135 mg/kg BW	Once a day for 5 consecutive days in adult (>2 months)	Nse-CreERT2; ROSA26-LSL-LacZ, Nse-CreERT2; ROSA26-tdTomato-flox-EGFP	
Aida et al. (2015)	25662838	IP	100 mg/kg BW	Once at P1	GLAST-CreERT2; GLT1-flox	Almost complete deletion of GLT1 protein.
Aida et al. (2015)	25662838	IP	100 mg/kg BW	Once a day for 5 consecutive days from P19	GLAST-CreERT2; GLT1-flox, GLAST-CreERT2; ROSA26-LSL-tdTomato	Reduction of GLT1 protein by 60–80%. Almost all astrocytes expressed tdTomato.
Aida et al. (2015)	25662838	IP	100 mg/kg BW	Once a day for 5 consecutive days from P84	GLAST-CreERT2; GLT1-flox	Mild reduction of GLT1 protein.
Huang et al. (2018)	30275311	IP	100 mg/kg BW	Once a day for 10 days (5 doses) every other day from 3 weeks old	Ubc-CreERT2, LSL-Rai1	Cre-dependent removal of stop casset before the start codon restored *Rai1* mRNA to control level.
(Diéguez-Hurtado et al., 2019)	31249304	IP	50 μg (25 mg/kg BW in 2 g mouse)	Once a day for 3 consecutive days from P1	Pdgfrb-CreERT2; ROSA26-mTmG	Recombination efficiency in cortical regions of the cerebrum was around 80% and no obvious differences were found among distinct regions of the brain.
(Diéguez-Hurtado et al., 2019)	31249304	IP	1 mg (111 mg/kg BW in 9 g mouse)	Once a day for 5 consecutive days from P21 and older	Pdgfrb-CreERT2; ROSA26-mTmG	Efficient targeting of mural cells was achieved in juvenile and adult animals.
(Ishii et al., 2021)	33443207	IP	75 mg/kg BW	Once a day for 3 consecutive days from P3	Ift88-flox;ER81-CreERT2; ROSA26-LSL-tdTomato	Robust tdTomato expression at P8 in layer V neurons.

Studies examining different doses of tamoxifen						
Reference	PMID	Route	Dose	Timing	Mouse line	Readout
Valny et al. (2016)	27812322	IP	200 mg/kg BW	Once a day for 2 consecutive days in P60-90 (for young adult)	C57BL6J (wild-type)	4-OHT concentration in brain is similar between 200 mg/kg and 100 mg/kg conditions
Valny et al. (2016)	27812322	IP	100 mg/kg BW	Once a day for 2 consecutive days in P60-90 (for young adult)	C57BL6J (wild-type)	
Jahn et al. (2018)	29651133	IP	100 mg/kg BW	Once at P28	C57BL/6N (wild type), GLAST-CreERT2; glia1-flox; p2ry1-flox, GLAST-CreERT2; ROSA26-LSL-tdTomato	Three injections increased time required for clearance of tamoxifen and it's metabolites than one injection. Five days of 100 mg/kg tamoxifen IP reaches almost 100% recombination in astrocytes in the cerebral cortex and cerebellum.
Jahn et al. (2018)	29651133	IP	100 mg/kg BW	Once a day for 2 consecutive days from P28	GLAST-CreERT2; glia1-flox; p2ry1-flox	
Jahn et al. (2018)	29651133	IP	100 mg/kg BW	Once a day for 3 consecutive days from P28	C57BL/6N (wild type), GLAST-CreERT2; glia1-flox; p2ry1-flox, GLAST-CreERT2; ROSA26-LSL-tdTomato	
Jahn et al. (2018)	29651133	IP	100 mg/kg BW	Once a day for 5 consecutive days from P28	GLAST-CreERT2; glia1-flox; p2ry1-flox	
Jahn et al. (2018)	29651133	IP	100 mg/kg BW	Once a day, three doses every other day from P28	GLAST-CreERT2; glia1-flox; p2ry1-flox	
Donocoff et al. (2020)	32943672	IP	6 mg (200 mg/kg BW in 30 g mouse)	Once a day for 5 consecutive days in 8–20 weeks old	CAGGCre-ERTM; ROSA26-LSL-EYFP	6 mg tamoxifen IP showed higher tamoxifen concentration in liver/spleen, and mortality
Donocoff et al. (2020)	32943672	IP	3 mg (100 mg/kg BW in 30 g mouse)	Once a day for 5 consecutive days in 8–20 weeks old	CAGGCre-ERTM; ROSA26-LSL-EYFP	
Donocoff et al. (2020)	32943672	PO	6 mg (200 mg/kg BW in 30 g mouse)	Once a day for 5 consecutive days in 8–20 weeks old	CAGGCre-ERTM; ROSA26-LSL-EYFP	
(Donocoff et al., 2020)	32943672	PO	3 mg (100 mg/kg BW in 30 g mouse)	Once a day for 5 consecutive days in 8–20 weeks old	CAGGCre-ERTM; ROSA26-LSL-EYFP	

Age at which tamoxifen is administered may affect recombination efficiency (Table 1). A single administration of 100 mg/kg BW of tamoxifen to GLAST-CreERT2 mice at P1 led to an almost complete deletion of the target protein, glutamate transporter 1. However, five daily administrations to P19 and adult mice led to a moderate or mild reduction, respectively (Aida et al., 2015). For neonatal mice, Pitulescu et al. intraperitoneally injected 50 μg tamoxifen for 3 consecutive days from P1 (equivalent to 25 mg/kg BW in a 2 g mouse), and 100 μg tamoxifen for 4 consecutive days from P5 (equivalent to 33 mg/kg BW in a 3 g mouse) (Pitulescu et al., 2010, 2017).

After preliminary experiments, we decided to administer 100 mg/kg BW tamoxifen once a day for five days from P28. For infants, we repeatedly injected 500 μg tamoxifen at P14, 17, 20 to minimum intervention of pup rearing. Given that pups weigh 6g, 500ug is equivalent to 83 mg/kg BW.

### Animals

All animal experiments must be approved by relevant institutional review board. The animal experiments described here were approved and conducted according to the guidelines established by the Institutional Animal Care and Use Committee of the University of Tsukuba.

We use *synapsin1*^*CreERT2*^ mouse line and *Sik3*^*ex13flox*^ mice in step-by-step protocol which were established in (Iwasaki et al., 2021). In *synapsin1*^*CreERT2*^; *Sik3*^*ex13flox*^ mice, tamoxifen administration induces exon 13 skipping allele of Sik3, which produces Sleepy (Slp) mutant SIK3. Male mice were used in this protocol. EEG/EMG recording were performed between 10- to 12-week-old.

To breed mouse lines, genotyping is required. The reagents and primer information used in (Iwasaki et al., 2021) are shown in key resources table. PCR cycling conditions are shown below. In genotyping of *synapsin1*^*CreERT2*^, the size of PCR products of wild-type allele amplified with synapsin1-CreERT2_C1 and synapsin1-CreERT2_W1 is 396 bp, and knock-in allele amplified with synapsin1-CreERT2_C1 and synapsin1-CreERT2_M1 is 443 bp. In genotyping of *Sik3*^*ex13flox*^, the size of PCR products of wild-type allele is 191 bp, and knock-in allele is 293 bp.

**Table:** PCR cycling conditions for genotyping *synapsin1*^*CreERT2*^

Steps	Temperature	Time	Cycles
Initial Denaturation	94°C	3 min	1
Denaturation	94°C	30 s	40 cycles
Annealing	60°C	30 s	
Extension	72°C	30 s	
Final extension	72°C	5 min	1
Hold	4°C	forever

**Table:** PCR cycling conditions for genotyping *Sik3*^*ex13flox*^

Steps	Temperature	Time	Cycles
Initial Denaturation	94°C	3 min	1
Denaturation	94°C	30 s	35 cycles
Annealing	60°C	30 s	
Extension	72°C	30 s	
Final extension	72°C	5 min	1
Hold	4°C	forever

***Alternatives:*** This protocol can be applied to other CreERT2 and flox mouse lines. We recommend that you observe the spatial and temporal expressions of the target gene using Allen Brain Atlas and in situ hybridization before generating new CreERT2 mouse lines. It is recommended to verify enough recombination efficiency is achieved and consider dose/timing of tamoxifen administration as needed. Please refer prior part “Tamoxifen dosage” for protocol modification. To show spatial recombination pattern in expected outcomes, we used *ROSA26*^*LacZ*^ mouse line in (Iwasaki et al., 2021).

***Alternatives:*** Adeno-associated virus (AAV)-based approaches are also possible to induce a mutant allele in the adult brain. For example, the local administration of Cre-expressing AAV in *Sik3*^*ex13flox*^ mouse brains can induce the exon 13-skipping allele. The local administration of a double-inverted orientation (DIO) of *Sik3 Slp (ex13-skipped)*-expressing AAV in an appropriate Cre mouse brains enables to express the mutant protein at a specific cell type.

### Preparation of materials required for EEG/EMG recording

We examined sleep/wake behavior using electroencephalogram/electromyogram (EEG/EMG) recording after CreERT2 induction. Specialized equipment is required such as recording chambers, stereotaxic apparatus, EEG/EMG electrodes, amplifiers and analog-to-digital converters, EEG/EMG analysis software. Protocol to make tether cable is shown below. Please refer to key resources table for equipment information.

**Timing: 1 day**

Preparation of tether cable

1. Cable preparation
  a. Prepare tools and materials shown in Figures 1A and 1B.

**Figure 1:**
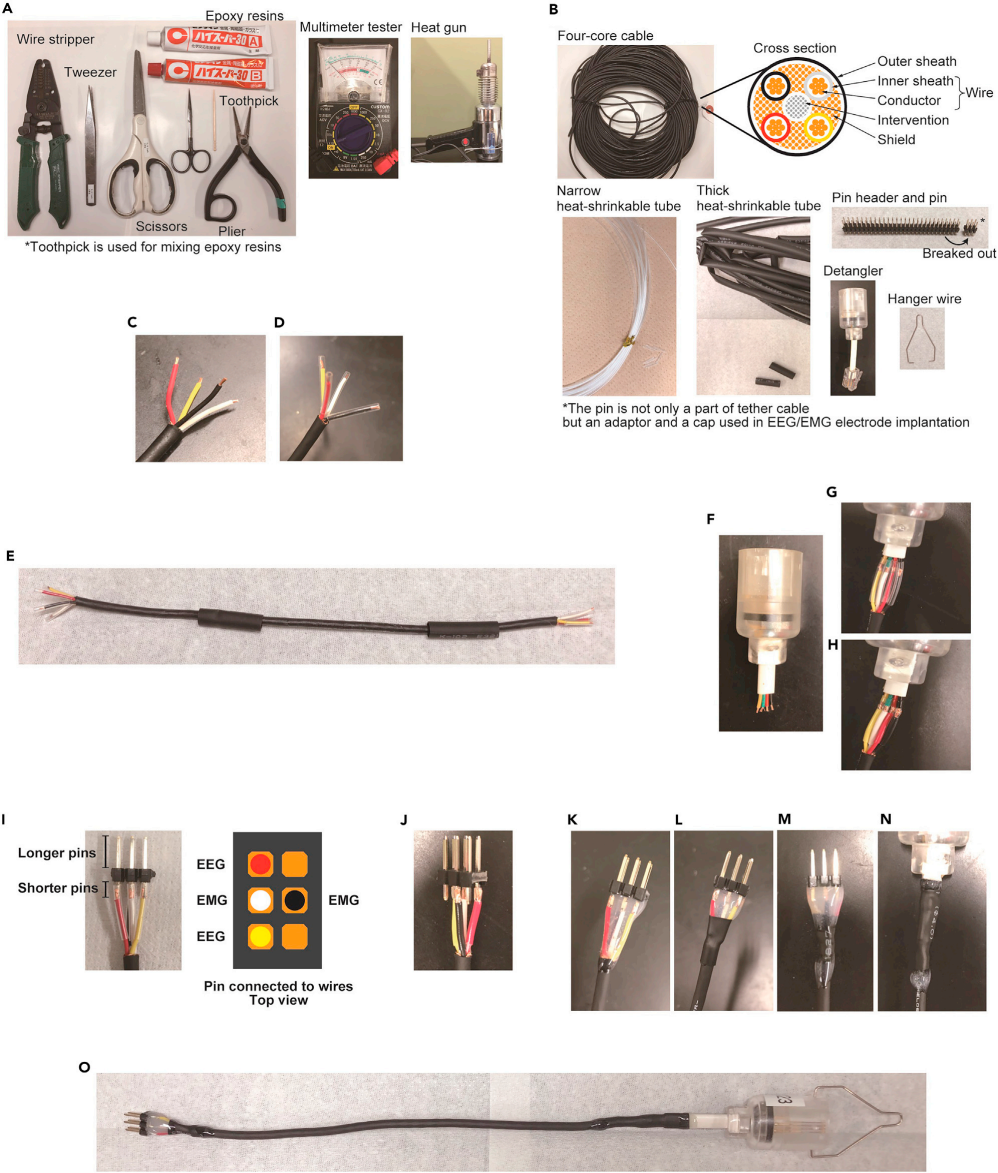
Tether cable preparation (A) Tools for tether cable preparation. (B) Materials of tether cable. (C) An end of four-core tether cable of which the intervention and shield are removed and inner sheath are stripped. (D) Each wire passing through narrow heat-shrinkable tubes. (E) Four-core cable assembled with 8 narrow heat-shrinkable tubes and 2 thick heat-shrinkable tubes. (F) Detangler cable of which inner sheath are stripped. (G) Conductors of detangler and four-core cable are in contact with each other in narrow heat-shrinkable tube. (H) Wires of detangler and four-core cable are connected after hot air is applied. (I) Shorter pins and conductors of four-core cable are in contact with each other in narrow heat-shrinkable tube. Allocation of EEG/EMG among pins are shown in schematic image. (J) Shorter pins and four-core cable are connected after hot air is applied. (K) Epoxy resin is applied from the outer sheath to the base of the electrode pins. (L) Thick heat-shrinkable tube covering the border between sheath and wires after sealing. (M) Epoxy resin is applied to the connections between heat-shrinkable tube and four-core cable, and heat-shrinkable tube to the base of the electrode pins. (N) Epoxy resin is applied to the connections between the detangler cable and heat-shrinkable tube, and heat-shrinkable tube and four-core cable. (O) A hanger wire is attached to the detangler. Finished tether cable.
  b. Cut the four-core cable into 17 cm pieces.
  c. Strip 1–1.2 cm of the outer sheath from both ends of the four-core cable with a stripper.
  d. Cut the metallic shields and intervention of the four-core cable from each end with small scissors
  e. Strip 1–1.2 mm of the inner sheath from each end of the four wires with a wire stripper (Figure 1C).
  f. Prepare eight thin heat-shrinkable tubes of 1 cm in length and two thick heat-shrinkable tubes of 2 cm in length (Figure 1B).
  g. Pass each wire through the thin heat-shrinkable tube (Figure 1D).
  h. Pass the cable through the two thick heat-shrinkable tubes (Figure 1E).
2. Detangler preparation
  a. Cut the detangler cable.
  b. Strip the outer sheath of the detangler cable from the end with small scissors.
  c. Strip 1–1.2 mm of the inner sheath from the four wires of the detangler cable using a wire stripper (Figure 1F).
3. Prepare electrode pins
  a. Bend the pin headers with pliers and cut it so that each piece has three pairs of electrode pins (Figure 1B).

4. Attach the four-core cable to the detangler cable
  a. Insert each detangler wire into the thin heat-shrinkable tube covering a specific wire (Figure 1G).
    **CRITICAL:** The conductors of the four-core cable and the detangler cable need to be in contact with each other.
  b. Apply hot air to the heat-shrinkable tubes with a heat gun to shrink them (Figure 1H).
    ***Note:*** Do not expose the thick heat-shrinkable tube to hot air.

5. Connect the four-core cable to the electrode pins to make a tether cable
  a. Insert each pin into the thin heat-shrinkable tube covering a specific wire (Figure 1I).
    **CRITICAL:** The conductors of the four-core cable and the electrode pins need to be in contact with each other.
  b. Apply hot air to the thin heat-shrinkable tubes with a heat gun to shrink them (Figure 1J).
    ***Note:*** Do not expose the thick heat-shrinkable tube to hot air.

6. Check energization
  a. Connect the tether cable to the multimeter tester with electrode pins and detangler.
  b. Make sure that the electrical resistance of all conductors are less than 20Ω and stable even when the detangler is twisted.
7. Fix connections with epoxy and attach hanger wire
  a. Fix the pins and cable connection with epoxy.
    i. Mix the two components of the epoxy resin.
    ii. Apply the mixed epoxy resin from the outer sheath to the base of the electrode pins and leave it until it hardens (Figure 1K).
    iii. Cover the border between sheath and wires with thick heat-shrinkable tube and seal it (Figure 1L).
    iv. Apply the mixed epoxy resin to the connections between heat-shrinkable tube and four-core cable, and heat-shrinkable tube to the base of the electrode pins. Leave it until it hardens (Figure 1M).
  b. Fix the detangler and cable connection with epoxy.
    i. Cover the connection from the four-core cable to the detangler cable with a thick heat-shrinkable tube and shrink it with a heat gun.
    ii. Apply the mixed epoxy resin to the connections between the detangler cable and heat-shrinkable tube, and heat-shrinkable tube and four-core cable. Leave it until it hardens (Figure 1N).
  c. Attach a hanger wire to the detangler (Figure 1O).

***Alternatives:*** There are several ways to assess sleep/wakefulness without EEG/EMG recording. However, EEG/EMG-based sleep analysis is the most reliable, especially for rapid eye movement sleep (REM sleep) and enables spectrum analysis that is required for the deeper analysis of sleep.

## Key resources table

**Table:** 

REAGENT or RESOURCE	SOURCE	IDENTIFIER
**Antibodies**		

Rabbit polyclonal anti-Sik3 C-terminus (1:2000)	(Funato et al., 2016)	N/A
Rabbit polyclonal anti-SIK3 ex13 (1:2000)	(Honda et al., 2018)	N/A
Rabbit polyclonal anti-Slp-specific SIK3 (1:2000)	(Iwasaki et al., 2021)	N/A
β-Tubulin (9F3) Rabbit mAb (1:1000)	Cell Signaling	2128
Peroxidase AffiniPure Donkey Anti-Rabbit IgG (H+L) (1:2000)	Jackson ImmunoResearch Laboratories	711-035-152

**Chemicals, peptides, and recombinant proteins**		

Tamoxifen	Sigma-Aldrich	T5648
Corn oil	FUJIFILM Wako Pure Chemical	032-17016
Corn oil (alternative)	Sigma-Aldrich	C8267
Isoflurane	FUJIFILM Wako Pure Chemical	099-06571
1M Tris-HCl pH7.6	Nacalai Tesque	35436-01
NaCl	Nacalai Tesque	31320-05
PhosSTOP	Roche Diagnostics	4906837001
0.5mol EDTA	Nacalai Tesque	06894-14
Phosphatase Inhibitor Cocktail2	Sigma-Aldrich	P5726
Protease inhibitor	TaKaRa	Z5673N
Sodium dodecyl sulfate	Sigma-Aldrich	L3771-100G
Sucrose	Nacalai Tesque	30404-45
Bromophenol Blue	FUJIFILM Wako Pure Chemical	029-02912
Trizma base (Tris)	Sigma-Aldrich	T1503-1KG
2-Mercaptoethanol	FUJIFILM Wako Pure Chemical	133-14571
Acrylamide	Nacalai Tesque	06114-95
Bisacrylamide	Nacalai Tesque	22407-52
Ammonium Persulfate (APS)	Nacalai Tesque	06284-04
TEMED	Nacalai Tesque	33401-72
Glycine	FUJIFILM Wako Pure Chemical	072-05285
HCl	FUJIFILM Wako Pure Chemical	080-01066
Tween 20	Sigma-Aldrich	P9416-100ML
Methanol	FUJIFILM Wako Pure Chemical	137-01823
BSA	Sigma-Aldrich	A6003-25G

**Critical commercial assays**		

Tissue Preparation Solution	Sigma-Aldrich	T3073-30ML
Neutralization Solution B	Sigma-Aldrich	N3910-24ML
RED Extract-N-Amp PCR Ready Mix	Sigma-Aldrich	R4775-125ML
Micro BCA assay	Thermo Fisher Scientific	23235
Clarity Western ECL Substrate	Bio-Rad Laboratories	170-5061

**Experimental models: Organisms/strains**		

Mouse: Syn1em1(cre/ERT2)Iiis or synapsin1CreERT2	(Iwasaki et al., 2021)	MGI:6506975
Mouse: Sik3tm2.1Iiis or Sik3ex13flox	(Iwasaki et al., 2021)	MGI:6506974

Oligonucleotides		

synapsin1-CreERT2_C1: GATCTGGAGGTGACCAGGAA	(Iwasaki et al., 2021)	N/A
synapsin1-CreERT2_M1: AACAAAGGCATGGAGCATCT	(Iwasaki et al., 2021)	N/A
synapsin1-CreERT2_W1: TGCCTCCACCTTGTCTCTCT	(Iwasaki et al., 2021)	N/A
Sik3-ex13flox_SLloxF: CTCTGACAGTTCTGTTCCAG	(Iwasaki et al., 2021)	N/A
Sik3-ex13flox_SLloxR: TGCCAGGAGAGTAGGCAGAT	(Iwasaki et al., 2021)	N/A

**Software and algorithms**		

LabVIEW	National Instruments	https://www.ni.com/en-us/shop/labview.html
MATLAB	MathWorks	https://www.mathworks.com/products/matlab.html

**Other**		

Syringe (29G)	Terumo	SS-05M2913
Four-core cable	Mogami wire	AWG28
Heat-shrinkable tubeφ0.6 mm	Woer Heat Shrinkable Material	suc-351
Heat-shrinkable tubeφ5.0 mm	Sumitomo Electric Fine Polymer	C5B
Detangler	SANWA SUPPLY INC.	TEL-TW2 (Discontinued)
Detangler (alternative)	Phillips	SJA4150
Pin header	Useconn Electronics	C-00082
Epoxy resin	Cemedine	CA-193
Heat Gun	ISHIZAKI ELECTRIC	PJ-214A
Multimeter tester	CUSTOM corporation	CX-02
Wire stripper	ENGINEER Inc	PA-14
Plier	HOZAN	P-51
Stereotaxic instrument	David Kopf Instruments	Model 940
Ear bars	David Kopf Instruments	Model 921
Drill Holder	David Kopf Instruments	Model 1474
Implant holder	David Kopf Instruments	Model 1770
Leutor (mortar unit)	Nihon Seimitsu Kikai Kosaku Co.	LGII M-22
Leutor (Power unit)	Nihon Seimitsu Kikai Kosaku Co.	LGII C-22
Leutor (Foot switch)	Nihon Seimitsu Kikai Kosaku Co.	FS-12
Tungsten carbide drill bit	SHOFU	HP-2
Anesthetic gas vaporizer	Shinano Seisakusho	SN-487-0T Air
Anesthesia induction box	Shinano Seisakusho	SN-487-85-03
Anesthetic gas absorber	Shinano Seisakusho	SN-487-61
Anesthetic gas scavenging system	Shinano Seisakusho	SN-489-4
Ethicon Needle with suture	Johnson & Johnson	W595
Dental cement	3M	RelyX Unicem 2
Surgery tools	Natsume Seisakusho	N/A
Multi-axis counter balanced lever arm	Instech Laboratories	MCLA
Hanger wire (as an accessory to the lever arm)	Instech Laboratories	MCLA
Recording cage	N/A	N/A
Recording cage top	N/A	N/A
Electrode	UNIQUE MEDICAL	(Miyoshi et al., 2019)
Preamplifier	Nihon Kohden	JB−641J
Amplifier	Nihon Kohden	AB-611J
AD converter	National Instruments	PCIe-6320
Vaseline	Kenei Phamaceutical	N/A
LED light	3M	Elipar S10
Silk thread	ETHICON	W595
Heating pad	Nissinrika	NHP-M30N
Liquid nitrogen	Cryogenics Div., Univ. of Tsukuba	N/A
Rotor-stator homogenizer	PRO Scientific	PRO200
PVDF membrane	Merck Millipore	IPVH00010
Chemiluminescence Imaging System	Vilber-Lourmat	Fusion Solo 6S.EDGE

## Materials and equipment

**Table:** Lysis Buffer

Reagents	Final concentration	Amount
Tris-HCl pH7.6 (1 M)	50 mM	50 μL
NaCl (1 M)	150 mM	150 μL
EDTA (0.5 M)	1 mM	2 μL
Phosphatase Inhibitor Cocktail2	1%	10 μL
Protease Inhibitor	1%	10 μL
MilliQ	n/a	Up to 1 mL
**Total**	**n/a**	**1 mL**

***Note:*** Prepare on ice before use.

**Table:** 6× Sample Buffer

Reagents	Final concentration	Amount
Sodium dodecyl sulfate (SDS)	12%	1.2 g
Sucrose	30%	3 g
Bromophenol Blue	0.03%	3 mg
Tris-HCl pH6.8 (1 M)	0.375 M	3.75 mL
MilliQ	n/a	Up to 7 mL
2-mercaptoethanol	30%	3 mL
**Total**	**n/a**	**10 mL**

***Note:*** Add MilliQ after the other reagents are dissolved by heating at 70°C for 15 min; Store the aliquot at −20°C without 2-mercaptoethanol and can be store for at least 6 months; Add 2-mercaptoethanol immediately before use and store at 4°C.

**Table:** 30% Acrylamide Mix

Reagents	Final concentration	Amount
Acrylamide	29.2%	14.6 g
Bisacrylamide	0.8%	0.4 g
MilliQ	n/a	Up to 50 mL
**Total**	**n/a**	**50 mL**

***Note:*** Store at 4°C protected from light, and do not store more than 6 months.

**Table:** Running Gel (7%)

Reagents	Final concentration	Amount
H_2_O	n/a	12.267 mL
30% Acrylamide Mix	7%	5.833 mL
Tris-HCl pH8.8 (1.5 M)	0.375 M	6.25 mL
SDS (10%)	0.1%	0.25 mL
Ammonium persulfate (APS)(10%)	0.15%	0.375 mL
TEMED	0.1%	25 μL
**Total**	**n/a**	**25 mL**

***Note:*** Add TEMED and mix immediately before pouring the gel into the mold.

**Table:** Stacking Gel (3%)

Reagents	Final concentration	Amount
H_2_O	n/a	5.912 mL
30% Acrylamide Mix	3%	0.8 mL
Tris-HCl pH6.8 (1 M)	0.125 M	1 mL
SDS (10%)	0.1%	80 μL
APS (10%)	0.25%	0.2 mL
TEMED	0.1%	8 μL
**Total**	**n/a**	**8 mL**

***Note:*** Add TEMED and mix immediately before pouring the gel into the mold.

**Table:** 10× Running Buffer

Reagents	Final concentration	Amount
Trizma base	250 mM	30.2 g
Glycine	1.92 M	144 g
SDS	1%	10 g
MilliQ	-	Up to 1 L
**Total**	**n/a**	**1 L**

***Note:*** Store at 18°C–24°C, and do not store more than 1 year. Dilute before use.

**Table:** 10× Transfer Buffer

Reagents	Final concentration	Amount
Trizma base	250 mM	30.2 g
Glycine	1.92 M	144 g
MilliQ	n/a	Up to 1 L
**Total**	**n/a**	**1 L**

***Note:*** Store at 18°C–24°C, and do not store more than 1 year. Dilute before use.

**Table:** 10× TRIS Buffered Saline (TBS)

Reagents	Final concentration	Amount
Trizma base	0.5 M	60.57 g
NaCl	1.5 M	87.66 g
HCl (12 M)	n/a	15–20 mL (adjust at pH7.6)
MilliQ	n/a	Up to 1 L
**Total**	**n/a**	**1 L**

***Note:*** Store at 18°C–24°C, and do not store more than 1 year.

**Table:** 1× TBST

Reagents	Final concentration	Amount
10× TBS	1×	100 mL
Tween 20 (10%)	0.1%	10 mL
MilliQ	n/a	Up to 1 L
**Total**	**n/a**	**1 L**

***Note:*** Store at 18°C–24°C, and do not store more than 2 weeks.

### Materials to confirm CreERT2 dependent recombination

#### Western blots using allele specific antibodies

In general, useful antibodies for detecting allele induction or deletion are as follows: 1) antibodies specific for sequences deleted by Cre-dependent allele induction/deletion; 2) antibodies specific for sequences encompassing adjacent exons after Cre-dependent allele induction/deletion. 3) antibodies specific for functional features such as amino acid sequence containing phosphorylated amino acids. Standard equipment for western blots is required. Please refer key resources table for product information that we used in the protocol.

#### Anti-SIK3 ex13 antibody

This rabbit polyclonal antibody was raised against LHAQQLLKRPRGPS using custom antibody production (Eurofins). The amino acid sequence is encoded by exon 13 and is expected to be less susceptible to phosphorylation because it is 8 amino acids away from 551 serine residue.

#### Anti-Slp-specific SIK3 antibody

This rabbit polyclonal antibody was raised against QLEYKAVPA spanning the sequence encoded by exon12 and exon 14 using custom antibody production (Eurofins). Resultant anti-serum was absorbed with QLEYKEQS (synthesized by Eurofins) spanning the sequence encoded by exon12 and exon 13 to eliminate the affinity for wild-type SIK3.

SIK3 sequence a part of exon12, 13, 14-encoded regions. QVAPNMNFTHNLLPMQSLQPTGQLEYK**EQSLLQPPTLQLLNGMGPLGRRASDGGANIQLHAQQLLKRPRGPSPLVTMTP**AVPAVTPVDEESSDGEPDQEA

Bold letters indicate amino acids encoded by exon 13 and RRAD (underlined) is the consensus sequence for PKA phosphorylation.

***Alternatives:*** Alternative antibody is required depending on the flox mouse line. Consider RT-PCR if there is no available antibody.

## Step-by-step method details

### Obtaining *synapsin1*^*CreERT2*^*; Sik3*^*ex13flox*^ mice

**Timing: More than 4 weeks**

*Synapsin1*^*CreERT2*^ and *Sik3*^*ex13flox*^ mice are crossed to obtain *synapsin1*^*CreERT2*^*; Sik3*^*ex13flox*^ mice. **Since the *synapsin1* gene is located on the X chromosome, the *synapsin1***^***CreERT2***^ **of males is passed from their mothers.** In order to increase efficiency, it is necessary to increase the number of mice with the appropriate sex and genotype. Young adult mice (2–4 months) are recommended when breeding starts.

1. Obtain homozygous *synapsin1*^*CreERT2*^ females. Following steps a and b shows how to obtain females with homozygous *synapsin1*^*CreERT2*^, which resides X chromosome, from a heterozygous mouse. When you do not have homozygous *synapsin1*^*CreERT2*^ females, please refer a and b.
  a. Cross *synapsin1*^*CreERT2*^ knock-in mice with wild-type mice of the opposite sex to obtain both heterozygous or homozygous *synapsin1*^*CreERT2*^ females and hemizygous *synapsin1*^*CreERT2*^ males.
  b. Cross a heterozygous *synapsin1*^*CreERT2*^ female and a hemizygous *synapsin1*^*CreERT2*^ male to obtain homozygous *synapsin1*^*CreERT2*^ females.
2. Cross homozygous *synapsin1*^*CreERT2*^ females and a heterozygous *Sik3*^*flox*^ male mouse to obtain male *synapsin1*^*CreERT2*^*; Sik3*^*ex13flox*^ mice.

**CRITICAL:** Appropriate regulations and guidelines for mouse experiments must be followed.

**CRITICAL:** The *synapsin1* gene is located on the X chromosome. Since the *synapsin1*^*CreERT2*^ of male mice is passed from their mothers, if you plan to use male mice for your experiment, heterozygous or homozygous *synapsin1*^*CreERT2*^ females are necessary to obtain hemizygous *synapsin1*^*CreERT2*^ males.

3. Check vaginal plugs every morning during breeding.
4. Transfer a pregnant female mouse to a separate cage in the case of polygamous mating.
5. Check daily to see if the female has given birth.
6. Record the date of birth.
7. Genotype the pups with Tissue Preparation Solution, Neutralization Solution B, and RED Extract-N-Amp PCR Ready Mix.

***Note:*** To see the effect of SIK3 SLP (exon 13-skipped) allele induction, we compared heterozygous *synapsin1*^*CreERT2*^ males with *Sik3*^*ex13flox*^ allele to those without *Sik3*^*ex13flox*^ allele (Iwasaki et al., 2021).

Littermates are recommended as control group because the genetic and environmental conditions can be identical except for the *Sik3*^*ex13flox*^ allele.

***Note:*** Please refer to the key resources table for reagents used in the genotype, and PCR conditions

8. Wean at around 4 weeks of age.

### Tamoxifen dilution

**Timing: 2 days**

Tamoxifen is diluted to 20 mg/mL in corn oil. Dilution should be started the day before the first IP injection. Dissolved tamoxifen should be used within 1 month in 4°C.

**CRITICAL:** Tamoxifen needs to be handled carefully according to safety data sheet because of the toxicity**.**

**Figure 2:**
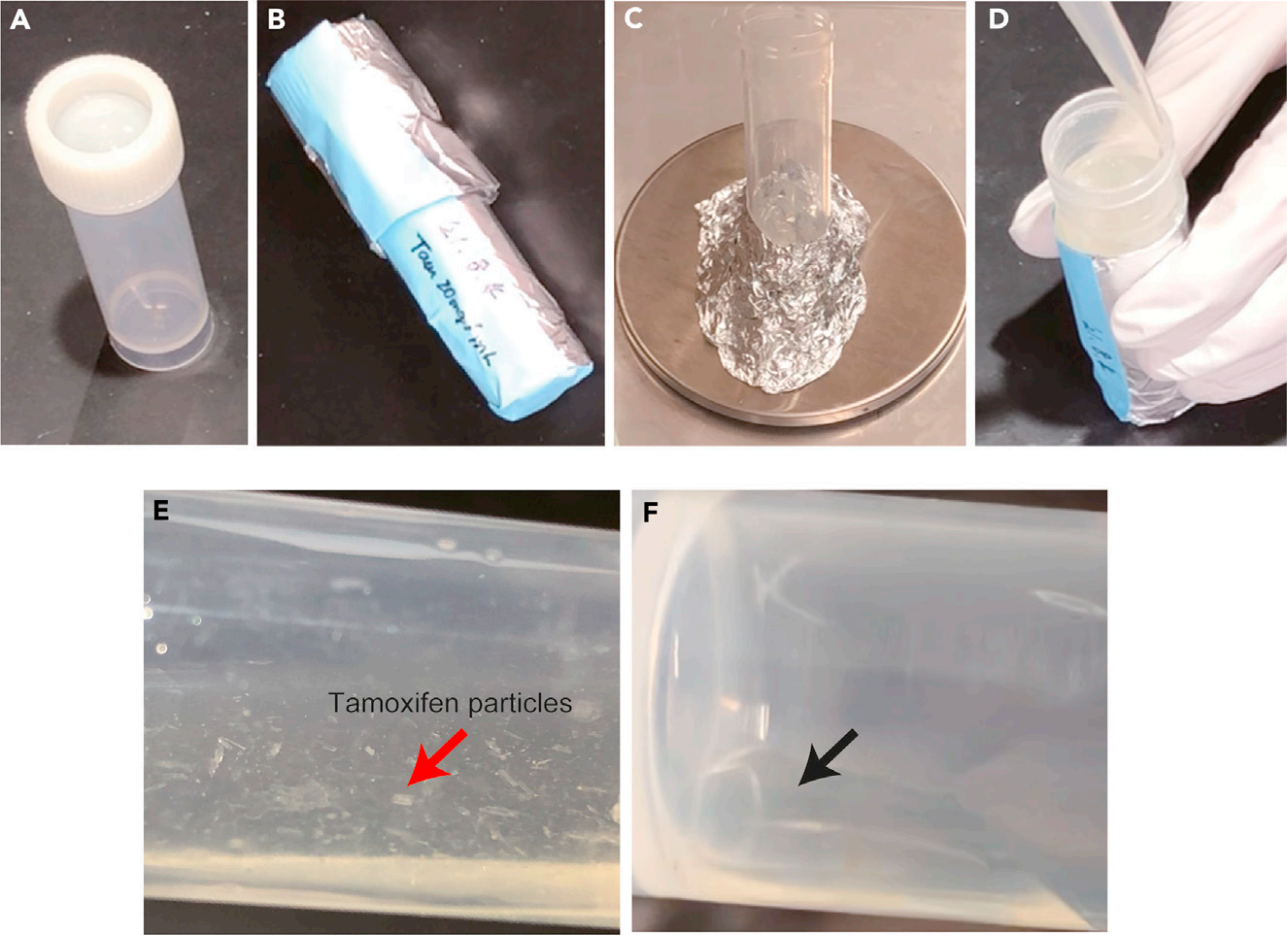
Procedure of dissolving tamoxifen in corn oil (A and B) Prepare a tube and cover it with aluminum foil. (C) Weigh tamoxifen in the tube prepared tube in (A and B). (D) Add warmed corn oil to tamoxifen. (E) Tamoxifen in corn oil before overnight incubation. (F) Corn oil in which tamoxifen is completely dissolved.
10. Bring tamoxifen to room temperature in a dark room and heat 5 mL of corn oil to 42°C.
11. Weigh 80 mg of tamoxifen in the tube prepared in step 10 (Figure 2C).
12. Add warmed 4 mL of corn oil to the tube and vortex (Figures 2D and 2E).

***Note:*** Change the amount of tamoxifen dilution as needed.

9. Cover a 15 mL-tube with aluminum foil to protect it from light (Figures 2A and 2B).

13. Incubate overnight (≒12 h) at 42°C with shaking.
14. Vortex several times during overnight incubation.
15. After incubation, vortex until tamoxifen is completely dissolved (Figure 1F).
16. Store at 4°C until use.

**CRITICAL:** Repeat vortex until no more tamoxifen particle are visible**.**

### Tamoxifen injection during late infancy

**Timing: 7 days**

Tamoxifen is delivered with IP injection

**Figure 3:**
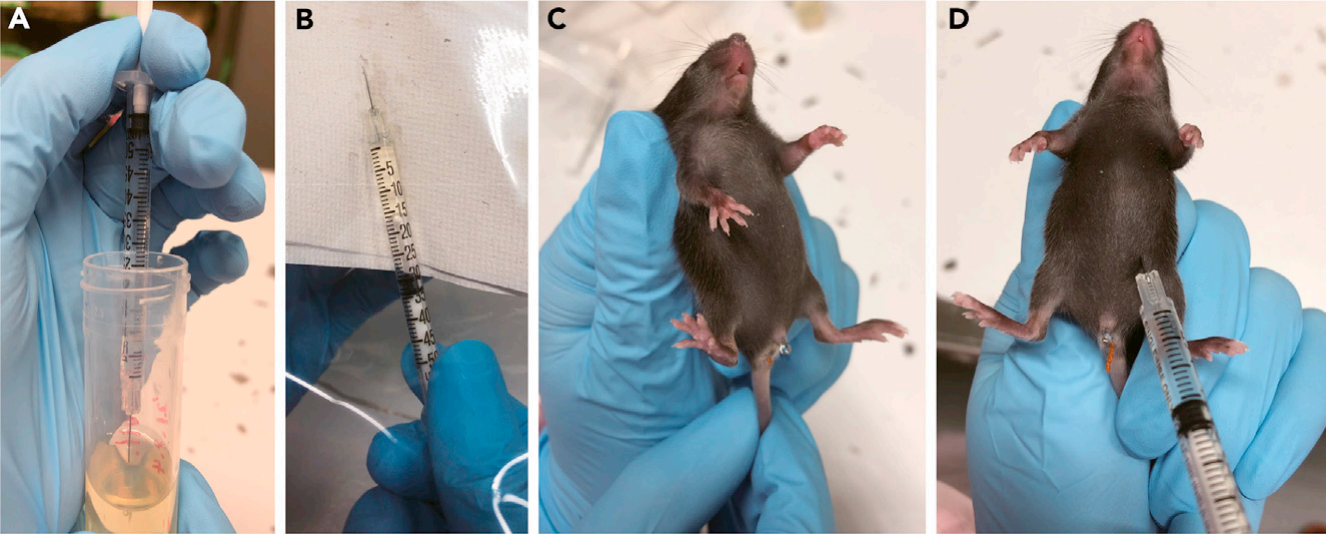
IP injection to P14 mice (A) Fill corn oil to syringe. (B) Remove air from syringe in plastic bag (to prevent scatter of tamoxifen and be discarded to medical pale). (C) Gently pinch pup’s back and keep tail between your fingers. (D) Insert the needle about 5 mm to the lower abdomen.
  b. Separate pups to a new cage.
  c. Gently stretch the pup along your hand by holding the back neck skin and tail (Figure 3C).
  d. Insert the needle to the lower abdomen towards the head at a 30° to horizontal. The needle was inserted only 5 mm (Figure 3D).
  e. Inject 50 μL of tamoxifen in corn oil intraperitoneally.
  f. Put the pups back to breeding cage.
    ***Note:*** This injection procedure is the same as giving an IP injection to an adult mouse.
    ***Note:*** It is recommended to fix injection time to avoid varying injection interval which may affect tamoxifen accumulation.
    ***Note:*** For histological examination, we sacrificed the mice 7–8 days after the last injection.

17. Dilute tamoxifen stock (20 mg/mL) 2 times with corn oil to 10 mg/mL.

18. Intraperitoneally inject 50 μL tamoxifen in corn oil (10 mg/mL) to pups on postnatal days 14, 17, and 20.
  a. Fill a syringe with the tamoxifen solution and remove air in syringe (Figures 3A and 3B).
    ***Note:*** Because of the high viscosity of corn oil, this step takes several minutes.

### EEG/EMG recording

**Timing: 2–3 weeks**

EEG/EMG electrode implantation surgery, EEG/EMG recording and analysis is performed (Iwasaki et al., 2021; Miyoshi et al., 2019). Here, we show the procedure of EEG/EMG implantation surgery. In our laboratory, EEG signals are recorded from ipsilateral metal pins on left hemisphere, and EMG signals are obtained from neck extensor muscle.

**Figure 4:**
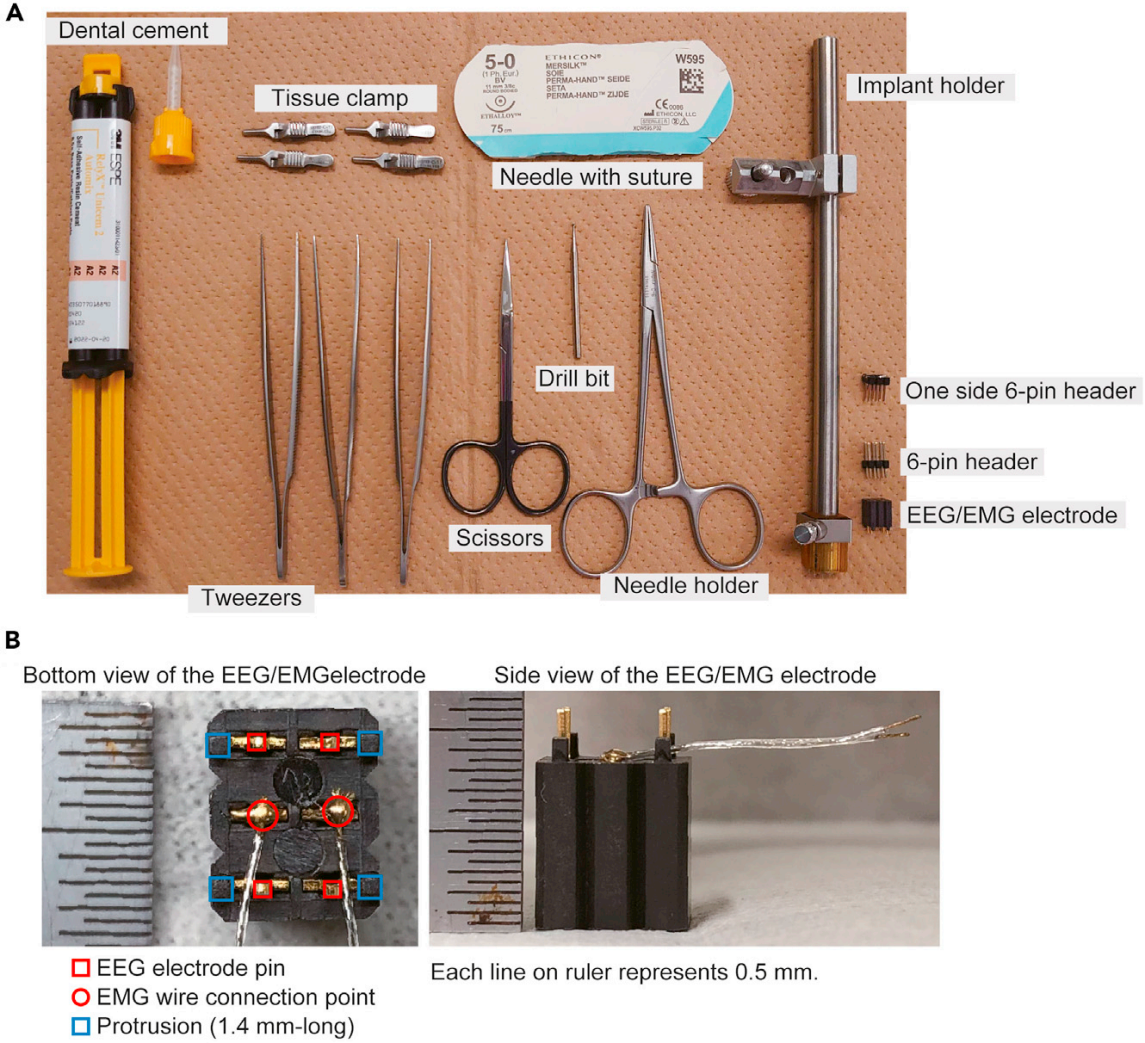
Tools for EEG/EMG electrode implantation (A) Tools for electrode implantation. One side 6-pin header is used as a “cap” for an EEG/EMG electrode after the implantation surgery. The bottom pins of 6-pin header are attached to an EEG/EMG electrode and the top pins are holed with implant holder during surgery. (B) Close-up pictures of EEG/EMG electrode.
  b. Place an 8–9-week-old mouse in an anesthesia box and anesthetize for 3 min with 4% isoflurane.
  c. Fix the mouse head in a stereotaxic instrument with two ear bars and a nose clamp. (Figure 5A).
    ***Note:*** Stable fixation capable of avoiding movement by drilling is required.

19. EEG/EMG electrode implantation
  a. Prepare tools for electrode implantation (Figure 4 and its figure legend).

**Figure 5:**
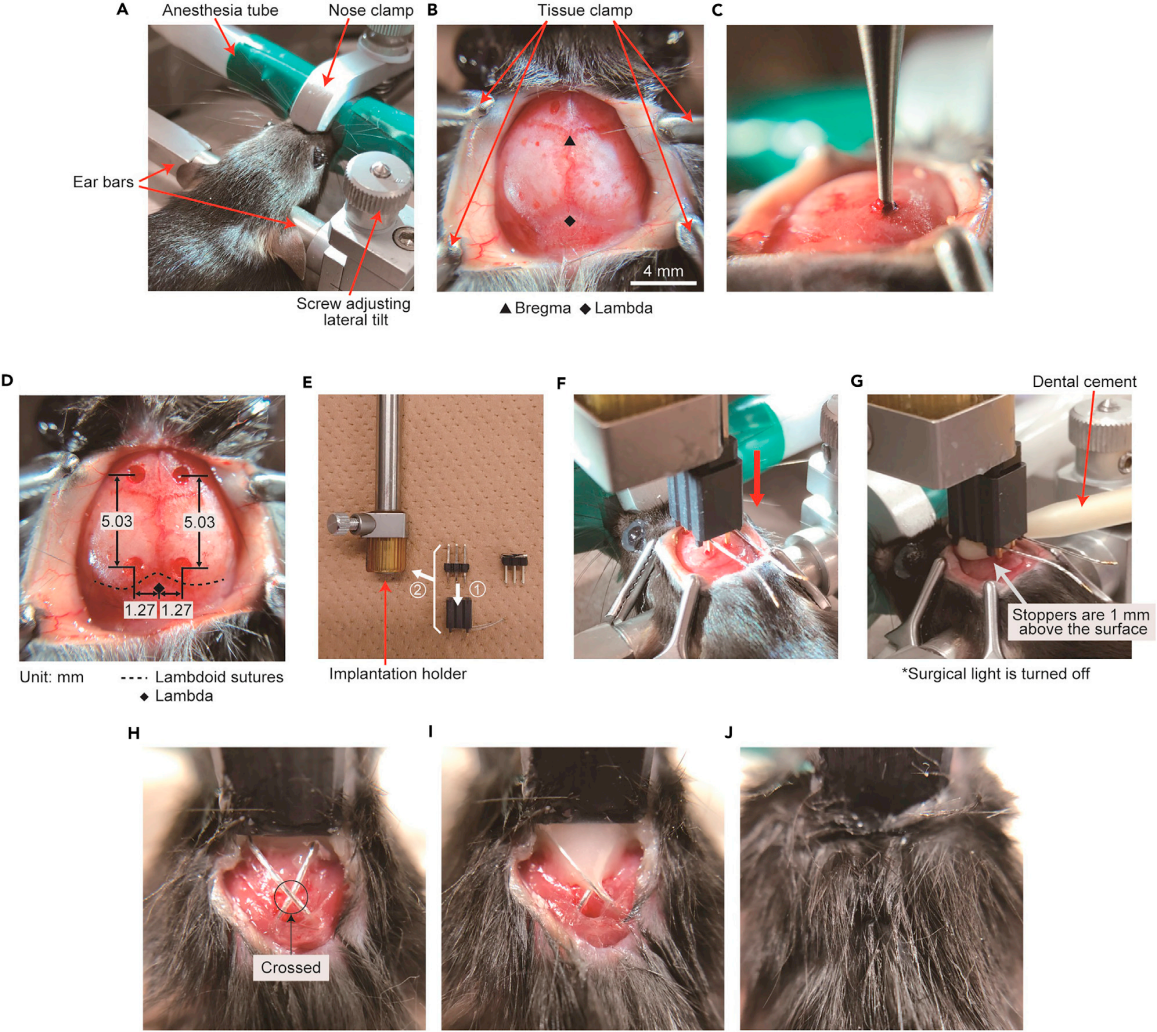
EEG/EMG electrode implantation (A) Head- fixed mice on stereotaxic apparatus. (B) Cranium surface is exposed. (C) Drilling 4 holes for EEG electrodes. (D) Drilling coordination. Posterior holes locate (x, y) = (± 1.27 mm, just anterior side of lambdoid sutures) and anterior holes locate (x, y) = (± 1.27 mm, 5.03 mm anterior from posterior holes). (E) How to fit electrode implantation to the holder. (F) Lower the electrode implantation. (G) Fill dental cement between skull surface and electrode implant. (H) EMG wires are inserted into neck extensor muscles. (I) EMG wires are fixed with dental cement. (J) Suture done.
  d. Decrease isoflurane concentration to 2%.
    ***Note:*** Observe respiratory rate and decrease isoflurane concentration if it is unstable or too slow.
  e. Apply Vaseline to the eyes, shave the fur from the top of the head, and disinfect the head skin with 70% ethanol.
  f. Make a longitudinal incision (2 cm long) along the midline of the scalp.
  g. Open the skin with four tissue clamps and clean the cranium surface with a cotton swab (Figure 5B).
  h. Adjust the head holder to set bregma and lambda at the same dorsoventral and mediolateral level (Figure 5B).
  i. Drill four holes in the skull for EEG electrodes and clean the skull surface with a cotton swab (Figures 5C and 5D).
    **CRITICAL:** Do not drill too deep so as not to damage the cerebral cortex. We use the tip of the drill reaching the outer surface of the skull as seen by the surgeon as a guide. But this varies depending on the drill used and needs to be adjusted for each researcher.
  j. Attach the electrode to the implant holder with long pins (made of a pin header, Figure 1) of the 6-pin header (Figure 5E).
  k. Lower the electrode pins into the holes under stereotaxic control until reach the four stoppers (small protrusion at the bottom of the implant to contact the skull) of the implant attach on the skull (Figure 5F).
    **CRITICAL:** Do not push the skull with the stopper.
    **CRITICAL:** Of the four stoppers, at least two on the left side should be attached to the skull for EEG recording.
    **CRITICAL:** When two stoppers on the left side is not attached to the skull because of displacement in horizontal direction of skull, correct the tilt with screws on the stereotaxic instrument.
  l. Set the Z axis to zero and heighten the Z axis of the implant to 1.0 mm.
  m. Apply dental cement between the skull and the electrode base (Figure 5G).
    **CRITICAL:** Turn off the surgical light as the dental cement is cured by strong light**.**
    **CRITICAL:** Make sure that the cement does not contain air when it is filled.
  n. Lower the Z axis of the implant to zero and cure the dental cement with LED light.
    ***Note:*** Remove cement adhered to the EMG wires
  o. Cross the two EMG wires and insert them into neck extensor muscles under the fascia (Figure 5H).
  p. Apply dental cement from the base of the wires to the intersection and cure the dental cement with LED light (Figure 5I).
  q. Apply two-four stitches using silk thread (Figure 5J).
    ***Note:*** Make sure there is no gap between the implant and the skin to prevent suture from being broken by the mouse.
  r. Stop the anesthesia and remove the mouse from the stereotaxic instrument.
  s. Remove the 6-pin header from the implant and then insert a one side 6-pin header (Figure 6A).
    ***Note:*** Hold the implant, not the mouse head, when removing the 6-pin header and inserting the one side 6-pin header.

**Figure 6:**
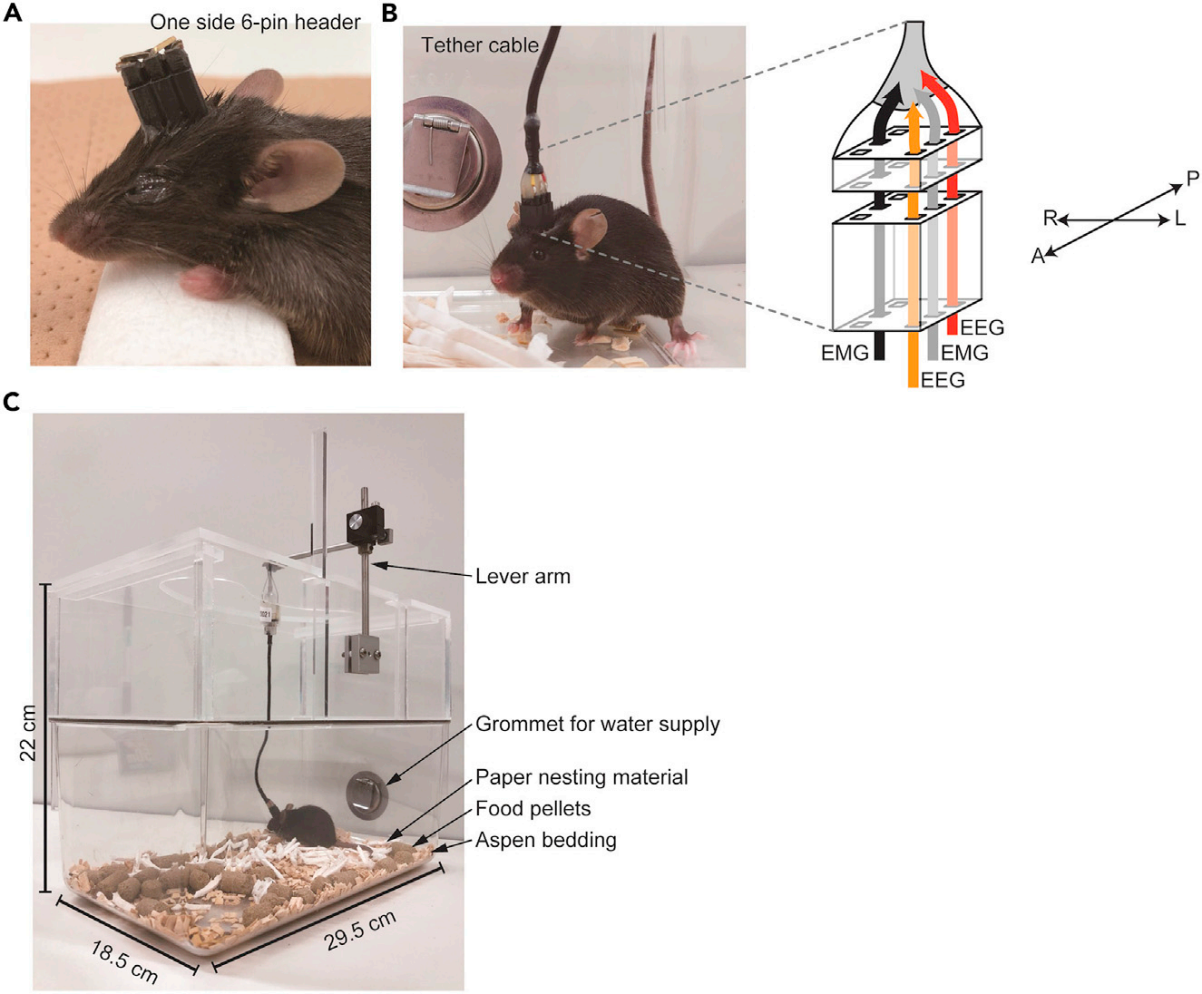
Mouse after the surgery (A) The mouse released from stereotaxic apparatus. A one side 6-pin header is inserted in place of the 6-pin header with long pins. (B) The mouse with tether cable in a recording cage. Tether cable is connected as shown in right schematic image. A, anterior; P, posterior; R, right; L, left. (C) Recording environment. The tether cable is hanged by a multi-axis counter balanced lever arm.
  t. Carefully wipe Vaseline from eyes.
  u. Place the mouse in the cage on the heating pad to keep it warm until the mouse starts normal behavior.

**Figure 7:**
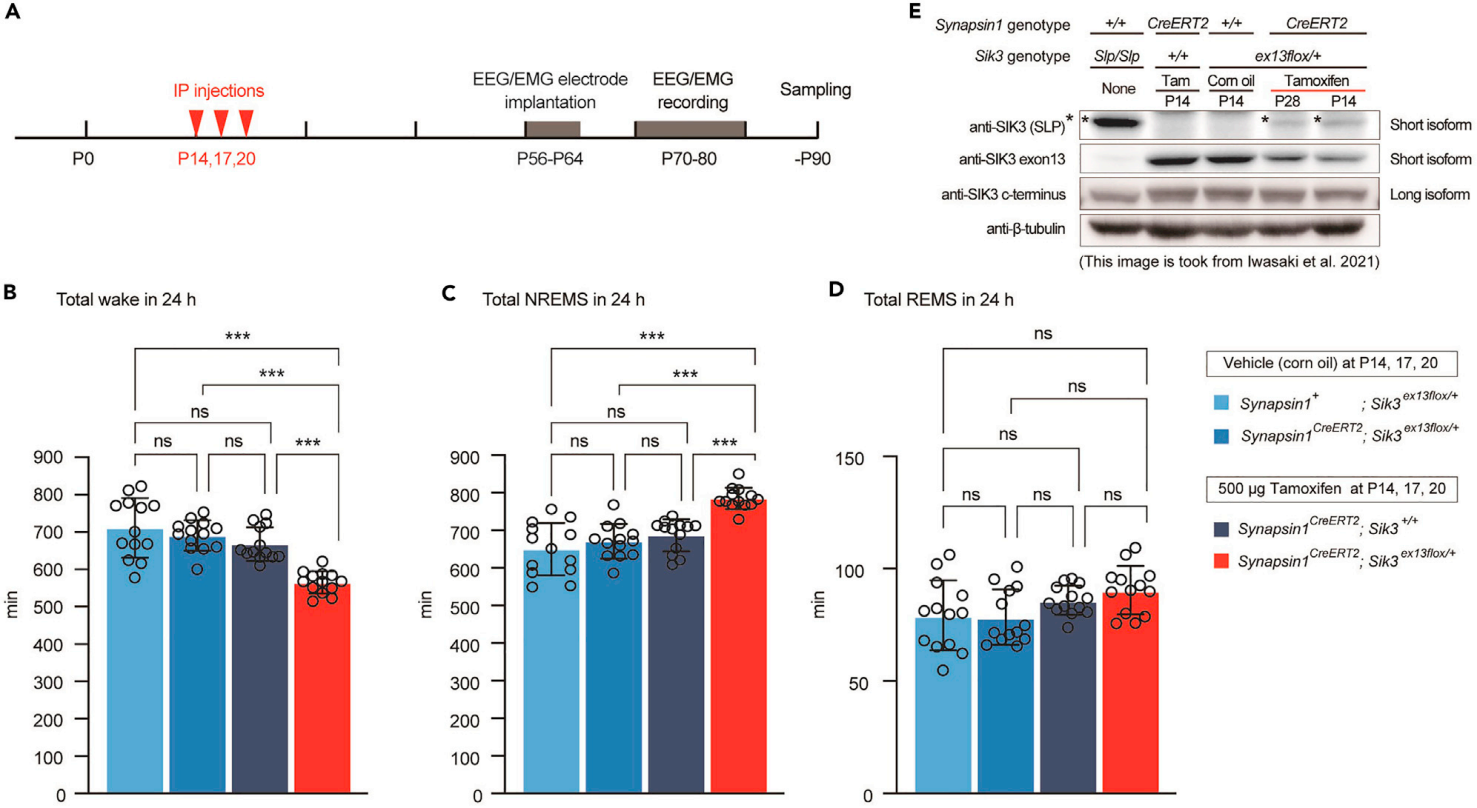
Sleep/wake behavior and mutant allele induction in tamoxifen administered synapsin1-CreERT2; Sik3-exon13 flox mice (A) Timeline for tamoxifen treatment followed by EEG/EMG recording. (B–D) Time spent in wake (B), NREM sleep (NREMS) (C), and REM sleep (REMS) (D) for 24 h in *synapsin1*^*CreERT2*^*; Sik3*^*ex13flox/+*^ mice that were administered tamoxifen or vehicle at P14, P17, and P20. One-way ANOVA followed by Tukey’s test; 13 mice per group. Data are mean ± SEM. ∗∗∗p<0.001. For more information, please refer to the original paper (Iwasaki et al., 2021). (E) Western blotting of brain homogenates. An antibody specific to ex13 skipping SIK3 detected the protein (asterisks) in tamoxifen administrated *synapsin1*^*CreERT2*^*; Sik3*^*ex13flox/+*^ mice and *Sik3*^*Slp/Slp*^ mice (top row). An antibody specific to the exon 13-encoded region detected wild-type SIK3 protein in tamoxifen- or vehicle-administered *synapsin1*^*CreERT2*^*; Sik3*^*ex13flox/+*^ mouse brains, but not in *Sik3*^*Slp/Slp*^ mouse brains (middle row). An antibody specific to SIK3 c-terminus detected long isoform of SIK3 (third row). In wild-type brains, there are two major isoforms of SIK3: short isoform (50–75 kDa, top and second row) and long isoform (150–250 kDa, third row) (Park et al., 2020). β-tubulin was used as a loading control (bottom row).
  d. Obtain and analyze EEG/EMG signaling with LabVIEW and MATLAB. Recording equipment are shown in key resources table. For more details of recording condition and analysis, please refer to Miyoshi et al. (2019) and Iwasaki et al. (2021).

20. EEG/EMG recording
  a. Allow the mouse to recover for at least 5 days in a home cage.
  b. Put the mouse in an anesthesia box (4% isoflurane) and wait until the mouse loses righting reflex. Take the mice from the anesthesia box and quickly remove the cap and attach a tether cable to the mouse.
  c. Acclimate the mouse to the recording environment for at least 7 days (Figures 7B and 7C).
    ***Note:*** Left side electrodes are used for EEG recording, so the electrode pins with three wires need to be left side (Figure 6B).
    ***Note:*** We wait at least 14 days from EEG/EMG electrode implantation to EEG/EMG recording.

### Western blotting for verification of recombination

**Timing: 3 days**

*Sik3* recombination was verified with western blotting. Mice were sacrificed after EEG/EMG recording for brain sampling.

21. Brain sampling for western blotting
  a. Prepare liquid nitrogen (LN2), tubes and dissection tools.
  b. Quickly dissect the brain after cervical dislocation and freeze it in LN2.
  c. Store at −80°C until homogenization.

**Pause Point:** frozen brain can be stored at −80°C for at least 3 months.

22. Western blotting
  a. Homogenize the brains using a rotor-stator homogenizer in ice-cold Lysis Buffer (100 μL Lysis Buffer for 1 mg brain).
  b. Rotate 15 min at 4°C for 30 min.
  c. Centrifuge at 17800×*g* at 4°C for 12 min.
  d. Immediately aliquot supernatant.
  e. Determine protein concentration with micro BCA assay.
  f. Dilute a part of supernatant with 6× Sample Buffer for SDS-PAGE and store the diluted sample and remaining sample at −80°C.
    **Pause Point:** frozen supernatants can be stored at −80°C.
  g. Perform SDS-PAGE with 7% Running Gel (under the 3% Stacking Gel layer) with 50 μg of protein in 1× Running Buffer.
  h. Activate PVDF membrane with methanol for 10 min at room temperature (18°C–24°C).
  i. Transfer the protein to PVDF membranes in 1× Transfer Buffer.
    ***Note:*** The concentration of acrylamide gel and protein concentration can be modified depending on your purpose.
  j. Wash the membranes in TBST and incubate overnight (12–18 h) at 4°C with a primary antibody in TBST with 5% BSA.
  k. Wash in TBST and incubate with HRP-conjugated for 2 h at room temperature (18°C–24°C), donkey anti-rabbit IgG (1:2000 dilution in TBST with 5% BSA; Jackson ImmunoResearch Laboratories)
  l. Wash in TBST and expose the blots to Clarity Western ECL Substrate.
  m. Detect chemiluminescence signaling with FUSION Solo 6S.EDGE.

## Expected outcomes

By following the protocol described here, we analyzed sleep/wake behavior of mice expressing SIK3 lacking exon 13 encoded region in neurons after late infancy (Figure 7A). Total wake time was decreased and NREM sleep time was increased in tamoxifen administered *synapsin1*^*CreERT2*^*; Sik3*^*ex13flox/+*^ mice (Figures 7B and 7C), but REM sleep time remained unchanged (Figure 7D). These result shows that *CreERT2* knock-in to *synapsin1* locus did not affect total time spent in each stage. Western blotting showed that SIK3 lacking exon 13 expressed in *synapsin1*^*CreERT2*^*; Sik3*^*ex13flox/+*^ mice only with tamoxifen administration (Figure 7E, Iwasaki et al., 2021).

## Limitations

The target exon that encodes functionally relevant amino acid sequence such as protein kinase recognition sequences is in-frame. Since the expression of *synapsin 1* is not evenly expressed in all neurons in the brain and tend to be more broadly expressed in earlier stage as shown (Iwasaki et al., 2021), the injection schedule of tamoxifen needs to be optimized.

## Troubleshooting

### Problem 1

Tamoxifen does not dissolve in corn oil (step 15).

### Potential solution

Increase the frequency of vortex during overnight incubation. Many protocols state that tamoxifen is dissolved at 37°C, but in our experience, that doesn't dissolve very well. Then, we set the temperature to 42°C to make it easier to dissolve. Please see Figures 2E and 2F.

### Problem 2

Mice are dead after tamoxifen administration (step 18).

### Potential solution

We have rarely experienced death of mice after tamoxifen administration in the current protocol. However, if mice frequently die after tamoxifen administration, the route and timing of administration should be changed appropriately to maintain a balance between the efficiency of Cre-dependent recombination and the survival rate. Please refer “Tamoxifen doseage” in this protocol for more information.

### Problem 3

Bleeding from the skull hole during the implantation surgery (step 19. i).

### Potential solution

Apply a clean twisted paper (kimwipes) into the skull hole and absorb the blood. In case of a lot of bleeding, apply pressure with a cotton swab against the skull hole to stop the bleeding.

### Problem 4

The implant dislodged from the skull after the implantation surgery (step 19. k).

### Potential solution

Before applying dental cement, carefully remove the blood and tissues on the skull with cotton swab.

### Problem 5

The tether cord restricts mouse’s movement since the mouse is small or weak (step 20. c).

### Potential solution

Using a thinner and softer tether will make it easier for the mouse to move. However, the tethers are weak, so always have spare tethers available, or change to a regular tether when the mouse grows.

### Problem 6

The clean EEG/EMG signals are not obtained (step 20. d).

### Potential solution

Replace the tether cable with a new one. During the implantation procedure, make sure that the two stoppers on the left side of the EEG are lightly touching the skull, the EMG wires are inserted into the neck extensors and are not touching each other, and that the electrode pins are clean.

## Resource availability

### Lead contact

Further information and requests for resources and reagents should be directed to and will be fulfilled by the lead contact, [Masashi Yanagisawa] (yanagisawa.masa.fu@u.tsukuba.ac.jp).

### Materials availability

This study did not generate new reagents.

## Data Availability

This study did not generate any data, a sophisticated custom computer code, or an algorithm.
